# Evaluation of Intraocular Pressure, Refraction, Anterior Chamber Depth, Macular Thickness, and Specular Microscopy Post-Neodymium-Doped Yttrium-Aluminum-Garnet Laser in Patients With Posterior Capsular Opacification

**DOI:** 10.7759/cureus.70987

**Published:** 2024-10-07

**Authors:** Radhika Paranjpe, Shreya Gandhi, Deepaswi Bhavsar, Khushboo Goyal, Tushar Agrawal, Kalpita B Goli

**Affiliations:** 1 Ophthalmology, Dr. D. Y. Patil Medical College, Hospital and Research Centre, Dr. D. Y. Patil Vidyapeeth (Deemed to be University), Pune, IND

**Keywords:** blindness, capsulotomy, cataract, intraocular pressure (iop), nd:yag laser, posterior capsule opacification (pco)

## Abstract

Background

Cataract is the leading cause of blindness globally, particularly in India. Despite advancements in surgical techniques, postoperative complications remain common, with posterior capsular opacification (PCO) being the most frequent issue. Although neodymium-doped yttrium-aluminum-garnet (Nd:YAG) laser capsulotomy is recommended for managing PCO, it is associated with various side effects. This study aimed to evaluate the effects of Nd:YAG laser capsulotomy on intraocular pressure (IOP), visual acuity, anterior chamber depth (ACD), macular thickness, and corneal endothelium in Indian patients.

Methodology

This prospective, hospital-based study was conducted in the ophthalmology department at a tertiary care center in western Maharashtra from September 2022 to June 2024. Approval from the Institutional Scientific and Ethics Committee was obtained before commencing the research. In the study, 72 eyes from 72 patients with PCO following uncomplicated cataract surgery who were undergoing Nd:YAG laser capsulotomy were included, whereas patients with corneal pathology, retinal pathology, complicated cataract surgery, or trauma were excluded. Patients with active uveitis, non-compliant patients, and those unwilling to undergo the procedure were also excluded. Written informed consent was obtained from each patient. Data were managed in Microsoft Excel, and statistical analysis was conducted using the SPSS 26.0 software. As the continuous variables exhibited skewed distribution, the Wilcoxon test was employed to assess categorical variables such as the significance of IOP and endothelial cell differences over time. A significance level of 5% was assumed, with a p-value below 0.05 considered significant.

Results

The mean age of patients who underwent Nd:YAG capsulotomy was 64 years, with a female predominance of 37 (51.4%). In the study, 37 (51.4%) patients had their left eye treated, while 35 (48.6%) had their right eye treated. Overall, 45 (62.5%) patients had a baseline best-corrected visual acuity (BCVA) of 6/24-6/12. At one hour post-procedure, 46 (63.9%) patients in Group II had a BCVA of 6/24-6/12, and by one week after treatment, 53 (73.6%) patients had a BCVA of >6/12-6/6. ACD was normal in all patients before and after the procedure. Two patients developed macular edema at one hour and one week after the procedure. The mean IOP at baseline, one hour, and one week were 13.5, 13.86, and 13.69 mmHg, respectively. A significant increase in IOP was observed at one hour post-procedure, along with a significant decrease in endothelial cell count compared to baseline, which also persisted at one week.

Conclusions

Patients undergoing Nd:YAG capsulotomy experienced an initial rise in IOP, followed by a subsequent decline. Improved visual acuity was noted in most patients at one hour and by one week. A significant decline in endothelial cell count was observed following the procedure, and macular edema was noted in two patients. Anterior chamber reaction was observed in nearly all patients, which decreased by one week. With no change in ACD following the procedure up to one week, ocular refraction was not significantly impacted in the short term. Therefore, further large-scale intervention studies are needed to evaluate the effects of Nd:YAG laser capsulotomy size and the energy used on refractive error and post-procedure complications, as well as to explore the long-term effects on IOP.

## Introduction

Globally, cataracts are the most prevalent cause of blindness [1], with India accounting for 66.2% of the blindness burden attributable to cataracts [2]. Surgery has proven to be an effective method for addressing this morbidity and improving vision [3,4]. However, despite recent advancements in cataract surgery, postoperative complications remain widespread, with the most common issue being posterior capsular opacification (PCO), which has multifaceted causes [5]. The primary causes of PCO are the movement and proliferation of residual lens epithelial cells (LECs) following cataract surgery, along with their transformation into fibroblastic and lens fiber-like cells. Younger individuals experience PCO more frequently and with greater severity than older patients due to a higher number of LECs and increased mitotic activity. Consequently, research is being conducted to develop safe and effective strategies to prevent PCO and improve surgical techniques [6].

PCO not only leads to quantitative visual disruptions but also degrades visual quality, resulting in glare, decreased contrast sensitivity, and loss of binocular vision [6]. It occurs in approximately 11.8% of cases within the first year, nearly 20.7% by three years, and 28.4% by five years, despite advancements in cataract surgical techniques and lens design [7]. The total prevalence can be as high as 50% in adults and 100% in children from two months to five years post-surgery [8]. Notably, PCO not only diminishes the patient’s quality of life but also complicates fundus and specialized ocular examinations, such as optical coherence tomography (OCT).

Currently, neodymium-doped yttrium-aluminum-garnet (Nd:YAG) laser posterior capsulotomy, which boasts a success rate exceeding 95%, is the standard treatment for PCO [9]. Laser capsulotomy creates a circular opening in the visual axis by applying a series of focused ablations to the posterior capsule using a quick-pulsed Nd:YAG laser [10].

The Nd:YAG laser is a solid-state laser that typically produces infrared light at a wavelength of 1,064 nm. When in pulsed mode, Nd:YAG lasers often employ Q-switching to generate high-intensity bursts, which can be frequency-doubled to produce light at 532 nm [11].

Nd:YAG laser capsulotomy is recommended for managing PCO that impairs visual acuity or function. Even in cases of minor capsular opacification, patients may still experience glare, making glare testing a useful tool for confirming these symptoms [12]. In India, the cost of the Nd:YAG laser procedure varies, reaching up to Rs. 40,000. The objective is to utilize the least amount of energy necessary to achieve the goal of breaking down and rupturing the capsule. Typically, most lasers can open a capsule using an energy setting of 1-2 mJ per pulse [12].

Although this procedure is effective, several complications may arise including a postoperative rise in intraocular pressure (IOP), inflammatory reactions, corneal burns, and macular edema, among others. Inflammatory mediators contribute to macular edema by damaging the blood-aqueous barrier, while elevated IOP is associated with an increased concentration of cells in the anterior chamber (AC) following Nd:YAG laser capsulotomy. Besides, retinal nerve fiber layer (RNFL) thickness may also be affected by elevated IOP. However, the increase in IOP is generally temporary [13]. Therefore, it is essential to understand the adverse event profile of the procedure in managing PCO within local settings.

Aim and objectives

This study aimed to observe the effects of Nd:YAG laser capsulotomy on visual acuity, IOP, refraction, anterior chamber depth (ACD), macular thickness, and corneal endothelium in pseudophakic patients with PCO. The objectives were to determine the side effects of the procedure and how it affected the aforementioned parameters one hour and one week following the procedure.

## Materials and methods

Study design

This prospective, hospital-based study was carried out between September 2022 and June 2024 at Dr. D. Y. Patil Hospital and Research Centre (DYPMCH), Pune, Maharashtra. A total of 72 eyes from 72 patients with PCO following uncomplicated cataract surgery who were undergoing Nd:YAG laser capsulotomy were included in the study, with each participant being subjected to a thorough clinical assessment and necessary investigations. The study received approval from the Institutional Ethics Committee of DYPMCH, Pune (approval number: IESC/PGS/2022/116).

Inclusion criteria

All patients presenting to the outpatient department of DYPMCH in western Maharashtra with previous uncomplicated cataract surgery and PCO undergoing Nd:YAG laser capsulotomy were included in the study.

Exclusion criteria

Patients with corneal pathology, such as endothelial dystrophies or degenerations, corneal scars, or trauma preventing good visualization, as well as those with any retinal pathology, active uveitis, or traumatic cataracts, were excluded from the study. Further, patients with complications from cataract surgery, such as vitreous loss, posterior capsular tears, or retinal detachment, as well as non-compliant patients and those unwilling to provide consent, were also excluded.

Sample size

The minimum sample size was calculated to be 72 using the Winpepi 11.38 software, considering 80% power and an acceptable difference of 10% with a 95% confidence interval [14].

Data and sample collection

A detailed ocular and systemic history was taken, including relevant past histories of ocular disease, trauma, or surgery. Moreover, a thorough history of cataract surgery was documented to rule out any complications, and Nd:YAG capsulotomy was performed after comprehensive history taking and examination. Then, a detailed evaluation was conducted before and after the procedure, which included measuring uncorrected visual acuity (UCVA) and best-corrected visual acuity (BCVA) for both near and distance vision using Snellen’s chart. Further, refractive power measurements were taken using an auto-refractometer, and ocular examinations of the orbit and adnexa, along with extraocular movements, were assessed. Next, a detailed slit-lamp examination of the anterior segment, including conjunctiva, cornea, AC, iris, pupil, and lens, was performed. Pupil dilation was achieved using tropicamide (0.08%) eye drops, and posterior capsular opacification was confirmed. Afterward, the posterior segment was evaluated using slit-lamp biomicroscopy with a 90 D Volk lens and indirect ophthalmoscopy with a 20 D Volk lens to rule out any pathology. IOP was measured using the Goldmann Applanation Tonometer, while OCT was performed for posterior segment evaluation, particularly for macular, RNFL thickness, and optic nerve head analysis. Finally, specular microscopy was conducted to evaluate the corneal endothelium.

Consent

In this study, the recruited patients were explained the study’s procedure and purpose. Thereafter, written informed consent was obtained from each patient.

Statistical analysis

Data management was conducted using Microsoft Excel (Microsoft Corp., Redmond, WA, USA), and statistical analysis was conducted using the SPSS 26.0 software (IBM Corp., Armonk, NY, USA). Frequency distributions and graphs were prepared for the variables. Further, quantitative data were summarized using mean (standard deviation, SD) and median (interquartile range, IQR), while qualitative data were summarized using proportions. As the continuous variables exhibited skewed distribution, the Wilcoxon test was used to assess categorical variables, such as the significance of IOP and endothelial cell differences over time. A significance level of 5% was assumed, with a p-value below 0.05 considered significant.

## Results

Table 1 presents the age-wise distribution of patients in the study population. Overall, 22 (30.6%) patients each belonged to the 51-60 and 61-70-year age groups.

**Table 1:** Age-wise distribution of patients.

Age	Frequency	Percent
40–50 years	6	8.3
51–60 years	22	30.6
61–70 years	22	30.6
71–80 years	21	29.2
81–90 years	1	1.4

Table 2 shows the baseline UCVA distribution among the study population. Overall, 49 (68.1%) patients had a baseline UCVA of 1/60-6/36, followed by 23 (31.9%) patients having a baseline UCVA of 6/24-6/12.

**Table 2:** Baseline UCVA distribution of patients. UCVA: uncorrected visual acuity

Baseline UCVA			Frequency	Percent
Group	I	(1/60-6/36)	49	68.1
Group	II	(6/24-6/12)	23	31.9
Total			72	100.0

Table 3 illustrates the baseline BCVA distribution among the study population. Overall, 45 (62.5%) patients had a baseline BCVA of 6/24-6/12, followed by 26 (36.1%) patients with a baseline BCVA of 1/60-6/36.

**Table 3:** Baseline BCVA distribution of patients. BCVA: best-corrected visual acuity

Baseline BCVA	Frequency	Percent
Group I (1/60-6/36)	26	36.1
Group II (6/24-6/12)	45	62.5
Group III (>6/12-6/6)	1	1.4
Total	72	100.0

Table 4 presents the UCVA distribution at one hour after treatment. An hour post-treatment, 58 (80.6%) patients were in Group II with a UCVA of 6/24-6/12.

**Table 4:** One-hour UCVA distribution of patients. UCVA: uncorrected visual acuity

UCVA (one hour)	Frequency	Percent
Group I (1/60-6/36)	14	19.4
Group II (6/24-6/12)	58	80.6
Total	72	100.0

**Figure 1:**
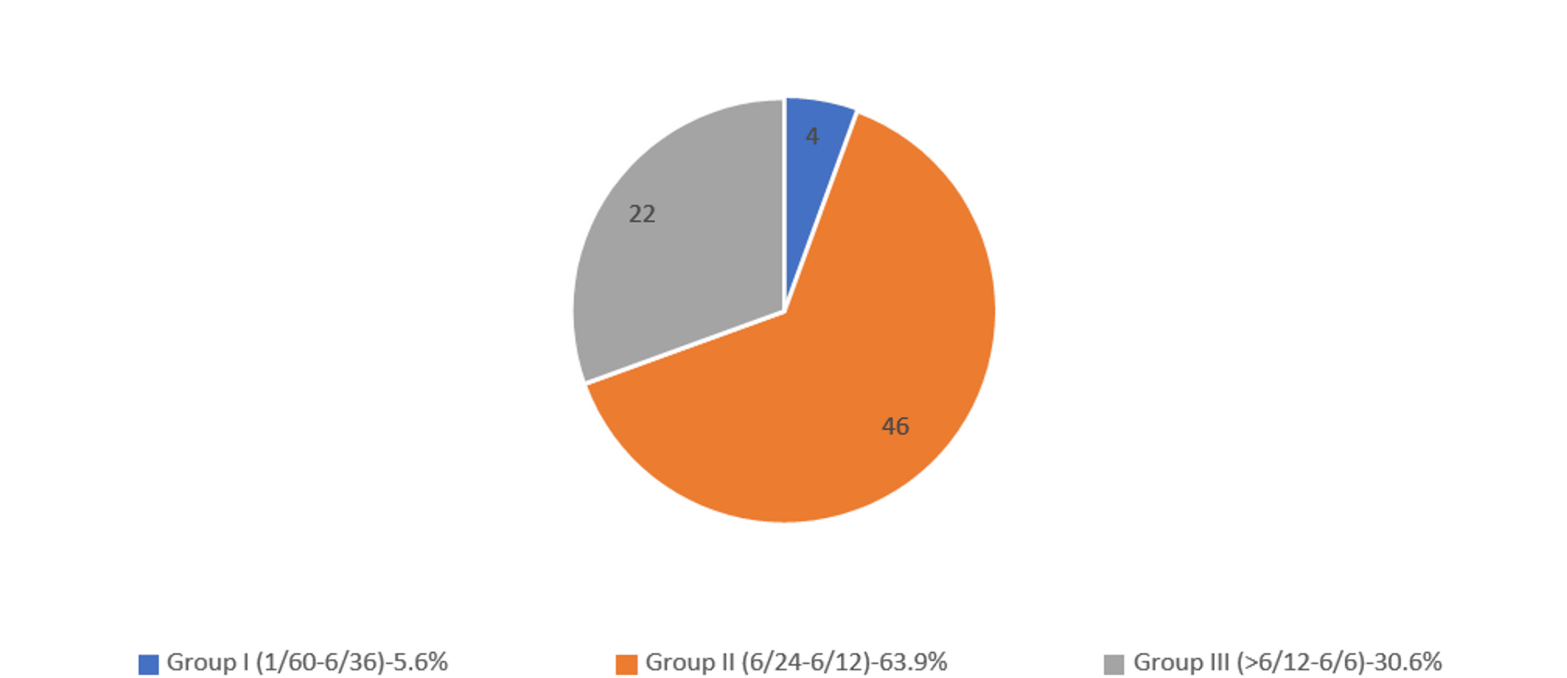
BCVA distribution (one hour after treatment). BCVA: best-corrected visual acuity

Figure 1 shows the BCVA distribution one hour after treatment. An hour post-treatment, 46 (63.9%) patients had a BCVA in Group II, i.e., 6/24-6/12, followed by Group III (>6/12-6/6) exhibited by 22 (30.6%) patients.

Table 5 shows the UCVA distribution at one week after treatment among the study population. A week post-treatment, 65 (90.3%) patients had a UCVA of 6/24-6/12, followed by 6 (8.3%) patients having a UCVA of 1/60-6/36.

**Table 5:** One-week UCVA distribution of patients. UCVA: uncorrected visual acuity

UCVA (one week)	Frequency	Percent
Group I (1/60-6/36)	6	8.3
Group II (6/24-6/12)	65	90.3
Group III (>6/12-6/6)	1	1.4
Total	72	100.0

Table 6 illustrates the BCVA distribution at one week after treatment among the study population. A week post-treatment, 53 (73.6%) patients belonged to Group III with a BCVA of >6/12-6/6, followed by 19 (26.4%) patients in Group II having a BCVA of 6/24-6/12.

**Table 6:** One-week BCVA distribution of patients. BCVA: best-corrected visual acuity

BCVA (one week)	Frequency	Percent
Group II (6/24-6/12)	19	26.4
Group III (>6/12-6/6)	53	73.6
Total	72	100.0

**Figure 2:**
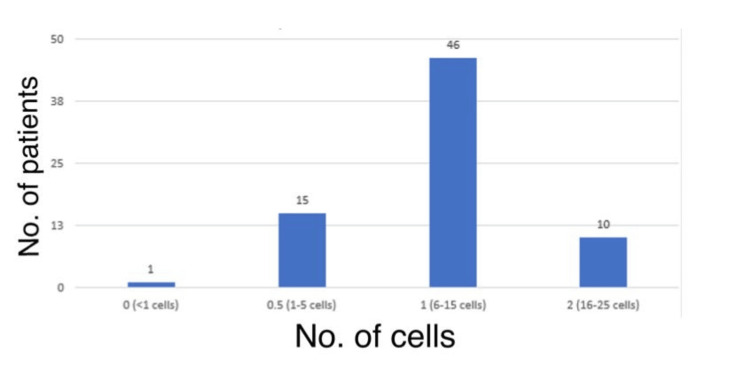
AC reaction (denoted by number of cells) at one hour. AC: anterior chamber

Figure 2 displays the distribution of AC reaction at one hour among the study population. The majority of the patients, i.e., 46 (63.9%), had an AC reaction of 1 (6-15 cells) at one hour. The ACD was normal in all patients at one hour and one week.

**Figure 3:**
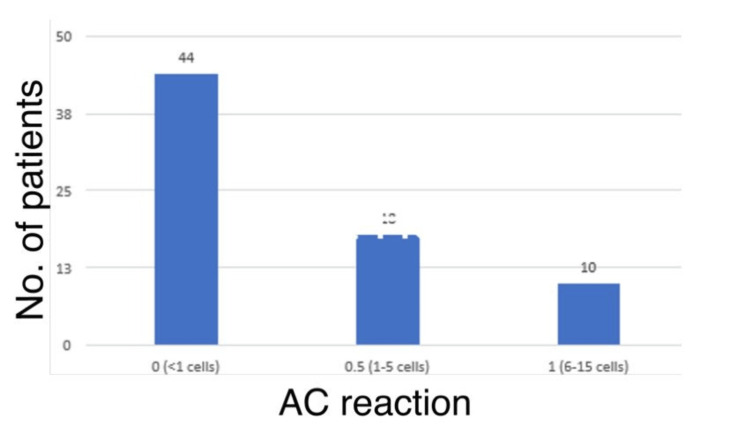
AC reaction at one week. AC: anterior chamber

Figure 3 shows the distribution of AC reaction at one week among the study population. Overall, 44 (61.1%) patients had an AC reaction of 0 (<1 cell) at one week.

Table 7 and Table 8 show the distribution of macular edema at one hour and one week among the study population. Two (2.8%) patients developed macular edema following the procedure at one hour and one week.

**Table 7:** One-hour macular edema distribution of patients.

Macular edema (one hour)	Frequency	Percent
Present	2	2.8
Absent	70	97.2
Total	72	100.0

**Table 8:** One-week macular edema distribution of patients.

Macular edema (one week)	Frequency	Percent
Present	2	2.8
Absent	70	97.2
Total	72	100.0

Table 9 presents the distribution of IOP in patients at baseline, one hour, and one week among the study population. The mean IOP at baseline, one hour, and one week was 13.5, 13.86, and 13.69 mmHg, respectively.

**Table 9:** IOP of patients at baseline, one hour, and one week. IOP: intraocular pressure; SD: standard deviation; IQR: interquartile range

IOP	Mean	Median	SD	IQR
IOP baseline	13.50	14.00	1.95	12, 14
IOP at one hour	13.86	14.00	2.11	12, 16
IOP at one week	13.69	14.00	1.95	12, 16

Table 10 illustrates the change in IOP at different time points from baseline. A significant increase in IOP was observed at one hour after the procedure compared to baseline. Although it fell below the one-hour values at one week, it did not return to baseline levels. The difference in IOP values remained significant at one week compared to baseline.

**Table 10:** Change in IOP at different time points from the baseline. IOP: intraocular pressure

IOP		N	Mean rank	Sum of ranks	P-value (Wilcoxon test)
IOP at one hour - IOP baseline	Negative rank	0	0	0	0.006
	Positive rank	9	5	45	
	Ties	63			
	Total	72			
IOP at one week - IOP baseline	Negative rank	0	0	0	0.008
	Positive rank	7	4	28	
	Ties	65			
	Total	72			

Table 11 shows the distribution of endothelial cell count in patients at baseline, one hour, and one week among the study population. The mean endothelial cell counts at baseline, one hour, and one week were 2488.43, 2487.85, and 2487.85, respectively.

**Table 11:** Endothelial cell counts of patients at various points. SD: standard deviation; IQR: interquartile range

Endothelial cell count	Mean	Median	SD	IQR
Endothelial cell count baseline	2,488.43	2,467.00	266.82	2,289.75, 2,687.75
Endothelial cell count at one hour	2,487.85	2,465.50	266.94	2,289.75, 2,687.75
Endothelial cell count at one week	2,487.85	2,465.50	266.94	2,289.75, 2,687.75

Table 12 presents the UCVA distribution in patients at baseline and one week after the procedure among the study population. In total, 43 (87.8%) patients with a baseline UCVA of 1/60-6/36 improved to 6/24-6/12 at one week after treatment.

**Table 12:** Change in UCVA of patients from the baseline till one week. UCVA: uncorrected visual acuity

UCVA * UCVA (one week)			UCVA (one week)			Total
			Group I (1/60-6/36)	Group II (6/24-6/12)	Group III (>6/12-6/6)	
UCVA baseline	Group I (1/60-6/36)	Frequency	6	43	0	49
		Percentage	12.2%	87.8%	0.0%	100.0%
	Group II (6/24-6/12)	Frequency	0	22	1	23
		Percentage	0.0%	95.7%	4.3%	100.0%
Total		Frequency	6	65	1	72
		Percentage	8.3%	90.3%	1.4%	100.0%

Table 13 shows the BCVA distribution in patients at baseline and one week after the procedure among the study population. In total, 12 (46.2%) and 14 (53.8%) patients with a baseline BCVA of 1/60-6/36 improved to 6/24-6/12 and >6/12-6/6 at one week after treatment, respectively. Additionally, 38 (84.4%) patients with a baseline BCVA of 6/24-6/12 improved to >6/12-6/6 at one week after treatment.

**Table 13:** Change in BCVA of patients from the baseline till one week. BCVA: best-corrected visual acuity

BCVA * BCVA (one week)			BCVA (one week)		Total
			Group II (6/24-6/12)	Group III (>6/12-6/6)	
BCVA baseline	Group I (1/60-6/36)	Frequency	12	14	26
		Percentage	46.2%	53.8%	100.0%
	Group II (6/24-6/12)	Frequency	7	38	45
		Percentage	15.6%	84.4%	100.0%
	Group III (>6/12-6/6)	Frequency	0	1	1
		Percentage	0.0%	100.0%	100.0%
Total		Frequency	19	53	72
		Percentage	26.4%	73.6%	100.0%

**Figure 4:**
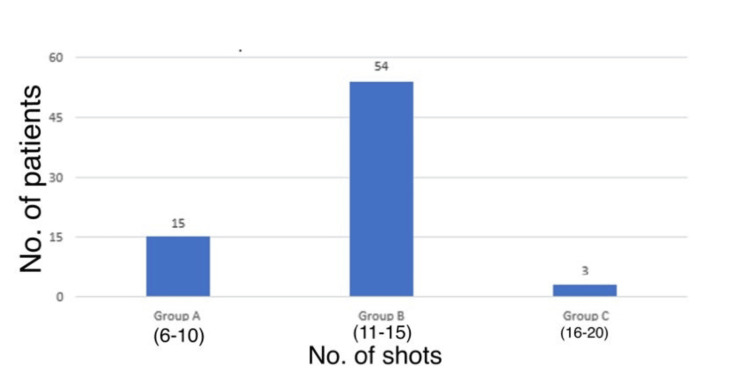
Distribution of the number of shots taken.

Figure 4 illustrates the distribution of the number of shots among patients included in the study population. Overall, 54 (75%) patients received 11-15 (Group B) shots.

## Discussion

In this study, the average age of the participants was 64.9 years. Overall, 22 (30.6%) patients each were in the age range of 51-60 and 61-70 years, followed by 21 (29.2%) patients in the 71-80-year age group, six (8.3%) in the 40-50-year age group, and one patient in the 81-90-year age group. In an experimental study of 120 PCO cases [15], patients had undergone small incision cataract surgery with posterior chamber intraocular lens implantation, and those on anti-glaucoma medications were excluded. Measurements such as BCVA, IOP, spherical equivalent, macular thickness, and ACD were taken in the preoperative, one-hour postoperative, and one-month postoperative periods. The mean age of patients in that study was 61.8 ± 7.69 years, which is lower than the present study (64 ± 9.9 years). Additionally, 62% were males in the previous study, whereas 35 (49%) males were evaluated in the present study [15].

In a retrospective, observational study [16], 68 pseudophakic eyes with PCO were treated with Nd:YAG following phacoemulsification. Patients were divided into two groups based on postoperative capsulotomy size (cut-off: 3.9 mm). The average age was 70.1 ± 7.8 years for the first group (<3.9 mm) and 71.5 ± 7.3 years for the second group (>3.9 mm). The energy usage for capsulotomy for the two groups was 57.6 ± 8.9 mJ and 61.0 ± 12.7 mJ, respectively. Another study [17] assessed visual acuity, IOP, and additional factors by examining two groups with varying average total energy levels (26.64 ± 12.92 mJ vs. 81.96 ± 32.1 mJ). The average age of participants was 62.72 ± 11.14 years in Group I and 60.68 ± 14.70 years in Group II. Moreover, the average age was similar to the present study. Notably, in most studies, the majority of patients belonged to the 61-70-year age group, which aligns with the present study.

In this study, most participants were females, i.e., 37 (51%), and the average age was 64, with an SD of 9.9 years. Additionally, 37 (51%) participants had their left eye treated while 35 (48.6%) had their right eye treated. Another study [18] evaluated changes in IOP following Nd:YAG for patients with PCO. Among the patients, 53% were males, and 49% were operated on the right eye. The average age of the pseudophakic category was 57 years. Age, gender, and eyes operated were comparable with the present study.

Moreover, in this study, baseline BCVA distribution indicated that 45 (62.5%) were in Group II (6/24 to 6/12), followed by 26 (36.1%) patients in Group III (>6/12-6/6), and one (1.4%) patient in Group III (6/12-6/6). One hour after the procedure, 46 (63.9%) patients were in Group II (6/24-6/12) and 22 (30.6%) in Group III (>6/12-6/6). By one week after the procedure, most patients, i.e., 53 (73.6%), were in Group III (>6/12-6/6), followed by 19 (26.4%) patients in Group II (6/24-6/12). A group of researchers [19] examined the structure and role of the corneal endothelium and visual acuity in individuals who underwent Nd:YAG laser capsulotomy. By one week, 90% achieved a visual acuity of >6/18, which increased to 94% by one month [19]. In another study [20], which evaluated the corneal endothelium and visual acuity following Nd:YAG laser capsulotomy using a specular microscope, 12% experienced a visual acuity improvement of 0.3 or less. In the present study, 45 (62.5%) patients were in Group II (6/24- 6/12), and by the end of the week, Group III (>6/12-6/6) had 53 (73.6%) patients. The percentage of patients achieving visual acuity of >6/12-6/6 increased from 1.4% (one patient) before the procedure to 73.6% (53 patients) by the end of the week, indicating better results in the present study at one week.

Furthermore, in the present study, 46 (63.9%) patients had an AC reaction of 1 (6-15 cells) at one hour, with the majority, i.e., 44 (61.1%), patients having <1 cell at one week, and the ACD was normal in all patients at one hour and one week. In the study by Khambhiphant et al. [21], the ACD increased from 3.74 to 3.79 in one week, but no significant difference was found. Changes in cylindrical refraction were noted one week later following capsulotomy, but these effects resolved in three months. However, the present study did not measure or compare these factors, nor did it record any changes in the position of the intraocular lens.

This study observed a prevalence of macular edema in two (2.8%) patients in the study population at one hour and one week. Macular edema following Nd:YAG laser may be attributed to disruption of the blood-aqueous barrier due to the influx of aqueous particles and inflammatory mediators. Raza [22] documented macular edema in 3% following Nd:YAG laser, while Ari et al. [23] determined that higher energy usage in laser is directly proportional to the impact on macular edema.

In the present study, the mean IOP increased from 13.50 at baseline to 13.86 at one hour before reducing to 13.69 at one week. In the study by Kaur et al. [15], the proportion of patients with visual acuity (>6/12-6/6) improved from 11.6% to 46%, and the mean IOP increased from 14.45 at baseline to 14.54 at one hour and 16.83 at two hours before subsequently declining to 16.04 at four hours and 15.11 at one day. By one week, the mean IOP was 14.58, and by one month, it decreased to 14.22. The mean IOP in the present study was lower than the findings of Kaur et al. [15], but the trend in IOP decline postoperatively was consistent across both studies. Additionally, in the study by Kaur et al. [15], the mean IOP in the group using >50 mJ was consistently higher than in the group using lower energy for the procedure. Hence, the difference in overall IOP could be attributed to the energy levels used during the procedure.

In the study by Karahan et al. [16], the IOP increased from a starting point of 15.44 ± 2.73 to 16.33 ± 2.42 one week later, then dropped to 15.13 ± 2.68 by one month, and continued decreasing to 14.83 ± 2.10 by the third month. However, the current research followed up for only one week and noted an increase from a starting point of 13.5 ± 1.9 to 13.9 ± 2.1 at one hour, which then fell to 13.7 ± 1.9 at one week. The variation in IOP levels at different time points could be attributed to the inclusion of an older age group in the study by Karahan et al. [16] in contrast to the present study. The early increase in IOP suggests that the dimensions of the Nd:YAG capsulotomy procedure play a significant role in outcomes, likely due to the release of inflammatory substances [16]. In the study by Parajuli et al. [17], IOP increased from 14.51 ± 2.53 to 14.98 ± 2.28 one hour after the procedure and then dropped to 14.64 ± 2.16 by the end of the month. The rate of increase in IOP was more pronounced in the first week for patients requiring more energy during the procedure. The follow-up period in the study by Parajuli et al. [17] indicated that IOP continued to decrease by one month.

In the study by Hassan [18], IOP was assessed at one hour, one day, and one week following the procedure. The average IOP before the procedure was recorded as 12.54 mmHg, which increased to 20.79 mmHg one hour post-procedure. Patients who experienced an IOP of 22 mmHg or higher at one hour were prescribed anti-glaucoma medications to control the rise in IOP. By the 24th hour, the IOP had decreased to an average of 13.24 mmHg in the groups. There was no significant variation in IOP changes between patients with clear corneas and those with artificial corneas. In the present study, the mean IOP increased from 13.50 ± 1.95 at baseline to 13.86 ± 2.1 at one hour before reducing to 13.69 ± 1.95 at one week. The decline in IOP over the postoperative period was consistent in both studies. However, the present study did not record the details of anti-glaucoma drug usage or its impact over time.

In a study involving 100 patients following capsulotomy [24], the changes in IOP observed in eyes that underwent capsulotomy were significantly more pronounced than in those that did not, and the increase in IOP was closely linked to the long-term elevation in IOP. Over time, IOP tends to be higher than pre-capsulotomy levels, especially in glaucoma patients or patients experiencing a significant spike in IOP shortly after the procedure. The present study noted a rise in IOP immediately after capsulotomy, which decreased by one week; however, the impact of this on glaucoma patients was not explored.

Lee et al. [25] estimated the effects of Nd:YAG laser on visual outcomes of patients operated on for cataracts. The effect was assessed at one week and four weeks post-Nd:YAG procedure. Patients were classified based on the time between phacoemulsification surgery and the Nd:YAG procedure. Those with a duration of less than one year were considered in the early Nd:YAG group, while those with more than one year were classified under the late Nd:YAG group. In the early Nd:YAG group, the IOP decreased from 16.22 ± 2.91 in the preoperative period to 16.15 ± 3.22 at one week and 16.07 ± 3.29 at one month. In the late group, the IOP increased from 15.67 ± 3.47 in the preoperative period to 15.74 ± 3.44 at one week and decreased to 15.21 ± 3.31 at one month. Although the IOP at each point was lower for the late Nd:YAG group, the IOP between the groups was not statistically different. In this study, the average IOP at one hour increased from 13.5 ± 1.9 at the start to 13.9 ± 2.1 and then dropped at one week to 13.7±1.9. The IOP was notably lower in this study than in the study by Lee et al. [25]. This variation could be attributed to the timing of the studies and advancements in technology over time. The current study followed up with patients for one week, and a comparison of IOP at one month could not be conducted. According to the findings of Icoz et al. [26], IOP did not show a notable change after the procedure. This may be attributed to the prophylactic use of anti-glaucoma medications given to all patients. Existing literature suggests that the use of anti-glaucomatous drugs reduces IOP [26].

In the current study, the average endothelial cell count declined from 2,488.43 ± 266.82 cells/mm^2^ at baseline to 2,487.85 ± 266.94 cells/mm^2^ one hour after the procedure, remaining the same at one week. In the study by Pathak et al. [19], the average endothelial cell density per square millimeter at baseline was 2,356.76 cells. However, this number decreased by one week to 2,231.8 cells/mm^2^ and by one month to 2,199.2 cells/mm^2^. A notable drop in endothelial cells was observed one hour after treatment compared to the baseline, which continued to be lower at one week. These results were consistent across both studies.

Nd:YAG capsulotomy was recorded to have several complications, including increases in IOP, damage to the lens, visual acuity changes, macular edema, retinal tears, and detachment. The most frequent complication reported was a rise in IOP. Therefore, without the use of anti-glaucoma medications, more than half of the patients experienced a rise of at least 10 mmHg after the procedure. Even with preventive measures, 15-30% of patients reported an increase in IOP in various studies [27].

Limitations

This study was limited to a single center, which may restrict the applicability of the findings to other settings and regions. The small sample size also limited the validity of its findings. Moreover, certain parameters, such as the energy used in the Nd:YAG laser procedure, were not collected in this study, although they were found to be significant in previous studies. We recommend changing the study design, including more cases, and showing laser settings (energy levels, number of shots, etc.) for future studies. Underlying medical conditions could act as confounders affecting recovery post-procedure and could not be quantified. Lastly, the relatively short follow-up duration (one week) did not allow for reporting long-term changes in the parameters.

## Conclusions

Patients undergoing Nd:YAG laser capsulotomy following uncomplicated cataract surgery with PCO experienced an initial rise in IOP, followed by a subsequent decline. Improved visual acuity was noted among most patients at one hour and by the end of one week. A significant decline in endothelial cell count was observed following the Nd:YAG capsulotomy, and macular edema was noted in two patients. Additionally, AC reaction was observed in almost all patients, which decreased by one week. With no change in ACD following Nd:YAG capsulotomy up to one week, the procedure did not significantly impact ocular refraction in the short term. Therefore, further large-scale intervention studies should be conducted to evaluate the effects of Nd:YAG laser capsulotomy size and the exact energy used on refractive error and post-procedure complications, as well as to explore the long-term effects on IOP.
